# Serum uric acid-to-albumin ratio as a novel predictor of all-cause mortality and cardiovascular events in hemodialysis patients: a retrospective cohort study

**DOI:** 10.3389/fnut.2026.1818356

**Published:** 2026-07-14

**Authors:** Qin Zhou, Qian Guan, Li Xu, Haitao Bai, Wenli Chen, Zeng Xingruo, Changxuan Liu

**Affiliations:** Department of Nephrology, The Central Hospital of Wuhan, Tongji Medical College, Huazhong University of Science and Technology, Wuhan, Hubei, China

**Keywords:** all-cause mortality, cardiovascular disease, hemodialysis, uric acid, uric acid-to-albumin ratio

## Abstract

**Background:**

Cardiovascular disease (CVD) is the leading cause of death in patients undergoing hemodialysis. The uric acid-to-albumin ratio (UAR), a novel marker, has been linked to cardiovascular diseases. However, its role in patients undergoing hemodialysis has not yet been studied. We aimed to investigate the association between UAR and the all-cause mortality and CVD events in hemodialysis patients.

**Methods:**

This single center retrospective cohort study was conducted between January 2021 and April 2024. Based on baseline UAR values, patients were categorized into tertiles. The associations between UAR and clinical outcomes were examined using Univariate and multivariate Cox regression models. Kaplan–Meier curves were used to estimate cumulative survival and CVD-free survival rates.

**Results:**

A total of 310 patients undergoing hemodialysis were included. During the 40-month follow-up period, 101 (32.6%) died, and 105 (33.9%) experienced new-onset CVD events. Kaplan–Meier survival curves showed significantly higher mortality rates in the middle and high UAR groups than in the low UAR group (Log rank= 10.684, *P*= 0.005). Patients in high UAR group were more likely to experience CVD events (Log rank= 14.006, *P*= 0.001). Multivariate Cox regression analysis revealed that, even after adjustment for traditional risk factors, high UAR remained significantly associated with an increased risk of all-cause mortality and new-onset CVD events (HR: 2.431, 95% CI: 1.407–4.200, *P* = 0.001; HR: 2.163, 95% CI: 1.304–3.588, *P* = 0.003; respectively).

**Conclusion:**

This study demonstrates for the first time that UAR is independently and significantly associated with all-cause mortality and CVD events in patients undergoing hemodialysis. These findings position UAR as a composite biomarker integrating inflammatory, metabolic, and nutritional pathways and provide a novel perspective for improving outcomes in patients undergoing hemodialysis.

## 1 Introduction

Cardiovascular disease (CVD) is the leading cause of death among patients receiving maintenance dialysis (1, 2). The mortality rate attributable to CVD in this population exceeds 50% (3). Inflammation and oxidative stress are closely associated with the development of CVD (3). Therefore, identifying appropriate mortality risk factors has become increasingly important to reduce cardiovascular events and improve patient survival.

The association between serum uric acid (SUA) levels and mortality risk in patients undergoing hemodialysis has been widely investigated. However, the relationship between SUA levels and long-term survival remains controversial. Some studies suggest that elevated uric acid levels are associated with an increased risk of death during dialysis (4–6). In contrast, other studies report that lower SUA levels are an independent risk factor for CVD, especially in those with low protein intake. This may be explained by the role of SUA as a potent antioxidant and nutritional marker, whereby higher SUA levels may reflect better nutritional status (7–9). Consequently, prognosis regarding mortality and CVD in patients on dialysis cannot be accurately predicted using uric acid alone. Serum albumin is a well-established indicator of nutritional status and possesses antioxidant and anticoagulant properties (10, 11). Lower serum albumin levels have been consistently associated with increased mortality risk in patients undergoing hemodialysis (12, 13). Recently, the uric acid-to-albumin ratio (UAR) has been shown to be associated with cardiovascular disease (14). As an inflammatory marker, UAR has been identified as an independent predictor of infarct-related artery thrombus (ICAT) burden in patients with ST-elevation myocardial infarction. Therefore, compared with a single indicator, UAR may produce better results as a novel predictor of all-cause mortality and cardiovascular events in patients undergoing hemodialysis by integrating nutritional status and oxidative stress.

To the best of our knowledge, the prognostic value of UAR for all-cause mortality and CVD events has not been previously investigated in patients undergoing hemodialysis. Accordingly, the purpose of this retrospective analysis was to evaluate the association between UAR and all-cause mortality as well as CVD events in this population.

## 2 Materials and methods

### 2.1 Subjects

This retrospective cohort study was conducted at the Dialysis Center of Wuhan Central Hospital. All patients received regular dialysis three times per week between January 2021 and April 2024. The inclusion criteria for this study were as follows: (1) age ≥18 years; and (2) patients who had received regular hemodialysis for more than 3 months. The exclusion criteria were: (1) malignancy; (2) transfer from peritoneal dialysis; (3) transfer from kidney transplantation; (4) acute kidney injury; (5) acute infection during the screening period; and (6) unavailable laboratory or follow-up data. Ultimately, 310 patients undergoing hemodialysis were included.

### 2.2 Ethics statement

This study protocol was reviewed and approved by the Medical Ethics Committee of the Wuhan Central Hospital (approval number: WHZXKYL2022-112-01). The participants provided written informed consent.

### 2.3 Data collection

Baseline clinical data included age, sex, body mass index (BMI), medical history (CVD and diabetes), and etiology of renal failure. Laboratory data for this study were obtained from the medical information system of Wuhan Central Hospital and included white blood cell (WBC) count, neutrophils, lymphocytes, monocytes, hemoglobin, serum albumin, serum creatinine, uric acid, serum phosphorus, corrected calcium, potassium, intact parathyroid hormone (iPTH), total cholesterol, triglyceride, high-density lipoprotein cholesterol (HDL-C), low-density lipoprotein cholesterol (LDL-C), C-reactive protein (CRP), and ferritin. All laboratory measurements were performed in the biochemical laboratory of Wuhan Central Hospital.

### 2.4 Clinical outcomes

The primary outcome of our study was all-cause mortality. The Secondary outcomes included new-onset CVD events during follow-up, such as peripheral vascular disease, acute coronary syndrome, congestive heart failure, transient ischemic attack, and stroke. Patients were followed until the end of the study (April 30, 2024).

### 2.5 Statistical analysis

Demographic and clinical characteristics were compared across UAR tertiles using appropriate statistical tests. Continuous variables with non-normal distributions were expressed as median (interquartile range) and analyzed using the Kruskal-Wallis test, whereas normally distributed variables were presented as mean ± standard deviation and analyzed using one-way analysis of variance (ANOVA). Categorical variables were summarized as frequencies (percentages) and compared using the chi-square or Fisher's exact test, as appropriate. Laboratory parameters and comorbidities were systematically analyzed to identify intergroup differences. Univariate Cox proportional hazards models were used to assess crude associations between clinical variables and study outcomes. Those factors without multicollinearity were selected for multivariate Cox regression analysis. Multicollinearity was assessed by using the variance inflation factor (VIF). VIF values greater than 5 indicated the presence of multicollinearity. Multivariate Cox proportional hazards models were constructed using forward selection (entry criterion: *P* < 0.10 in univariate analysis) and adjusted for clinically relevant confounders. Hazard ratios (HR) with 95% confidence intervals (CI) were calculated for continuous variables in their original measurement units and for categorical variables as dichotomous exposures. Kaplan-Meier survival analysis with log-rank testing was performed to compare cumulative event probabilities across UAR tertiles. All analyses were conducted using SPSS version 26.0 (IBM Corp.). The Bonferroni correction was used to correct the effects of multiple testing of statistical comparisons. A total of k=3 comparisons were performed, and statistical significance was accepted at a corrected threshold of a = 0.05/3 = 0.167. Another analyses was accepted with two-tailed *P* < 0.05.

## 3 Results

### 3.1 Baseline characteristics of the study population

**Figure 1:**
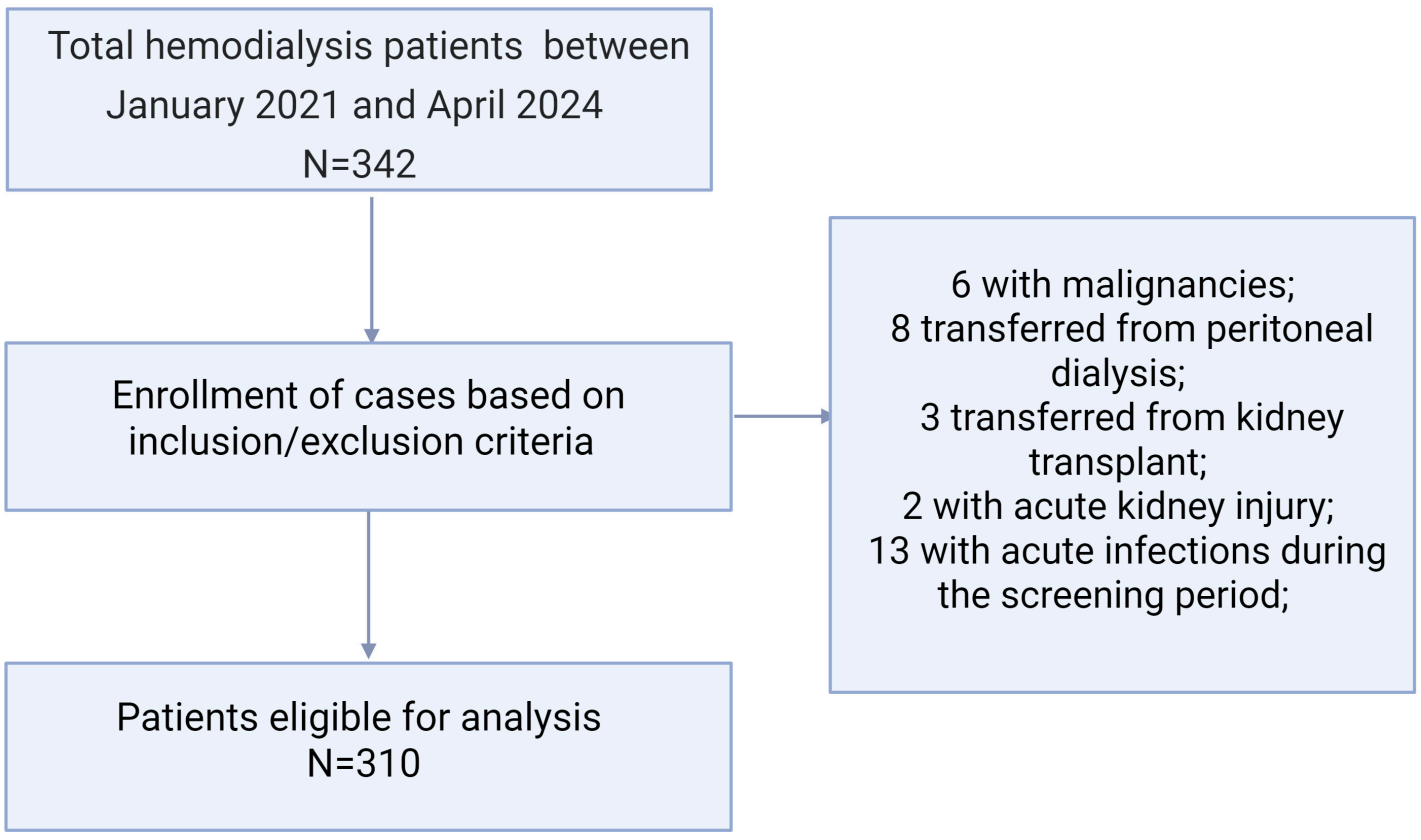
Study flow.

The baseline characteristics of the study participants are summarized in Table 1. A flowchart displaying patient selection is presented in Figure 1. A total of 310 patients undergoing hemodialysis were included. The median age was 66 years, and 59.4% (*n* = 184) were male. During the 40-month follow-up period, 101 patients (32.6%) died, and 105 patients (33.9%) experienced CVD events.

**Table 1:** Baseline characteristics of individuals stratified by tertiles of baseline UAR levels.

Variables	UAR levels				Bonferroni correction		
	Low (<8.70, *n* = 103)	Middle (≥8.70, <11.4, *n* = 104)	High (≥11.34, *n* = 103)	*P*-value	*P*-valueLvsM	*P*-value LvsH	*P*-value MvsH
Age (years)	66 (54, 74)	66 (57, 75.7)	67 (58, 73)	0.774	1.000	1.000	1.000
HD vintage (months)	46 (34–76)	35 (52–75)	54 (36–73)	0.862	1.000	1.000	1.000
Male (*n*, %)	58 (56.3)	63 (60.6)	63 (61.2)	0.616	0.473	0.479	0.608
BMI (kg/m^2^)	22.87 (20.61, 25.29)	22.21 (19.89, 25.20)	23.19 (20.39, 25.96)	0.244	1.000	1.000	0.614
Diabetes (*n*, %)	51 (49.5)	42 (40.4)	38 (36.9)	0.238	0.178	0.067	0.414
Hypertension (%)	102 (99)	100 (96.2)	101 (98.1)	0.366	0.275	0.561	0.516
Cerebrovascular disease (*n*, %)	20 (19.4)	19 (18.3)	18 (17.5)	0.937	0.833	0.719	0.882
Coronary artery disease (*n*, %)	48 (46.6)	48 (46.2)	43 (41.7)	0.741	0.879	0.483	0.617
ESRD causes				0.260	0.183	0.076	0.891
Glomerulonephritis (*n*, %)	9 (8.71)	15(14.42)	18 (17.48)				
Diabetic nephropathy (*n*, %)	39 (37.87)	27 (25.96)	23 (22.33)				
Hypertension nephropathy (*n*, %)	17 (16.50)	13 (12.50)	18 (17.48)				
obstructive nephropathy (*n*, %)	0 (0)	1 (0.01)	1 (0.01)				
Other/unknown (*n*, %)	38 (36.89)	48 (46.15)	43 (41.75)				
**Laboratory results**							
White blood cell count (× 109/L)	5.78 (5.78, 4.83)	6.05 (4.68, 7.34)	6.42 (5.50, 7.63)	0.102	1.000	0.146	0.166
Hemoglobin (g/L)	108 (93, 119)	113 (101.25, 124.75)	109 (94, 121)	0.028	0.025	0.564	0.545
Platelet count (× 109/L)	185 (137, 228)	175 (141.25, 213.75)	188 (146, 226)	0.694	1.000	1.000	0.979
Lymphocyte (× 109/L)	1.14 (0.86,1.52)	1.36 (0.99, 1.72)	1.39 (0.93, 1.69)	0.027	0.050	0.059	0.954
Monocyte (× 109/L)	0.37 (0.28, 0.47)	0.36 (0.27, 0.48)	0.38 (0.29, 0.46)	0.942	1.000	0.574	0.856
Glucose (mmol/l)	5.65 (4.95, 7.08)	5.55 (4.99, 6.98)	5.98 (4.92, 7.67)	0.665	1.000	1.000	0.325
Albumin (g/L)	41.8 (38.4, 45.4)	41.65 (37.55, 44.45)	38.5 (35.6, 41.3)	0.000	1.000	0.000	0.000
Uric acid (umol/l)	272.68 ± 68.76	411.37 ± 52.81	511.15 ± 75.43	0.000	0.000	0.000	0.000
Total cholesterol	3.55 (2.70, 4.19)	3.96 (3.24, 4.53)	3.62 (3.06, 4.52)	0.015	0.043	0.625	0.665
Triglyceride	1.11 (0.73, 1.93)	1.28 (0.83, 2.16)	1.33 (0.93, 2.02)	0.096	0.824	0.030	0.441
LDL (mmol/l)	1.88 (1.35, 2.43)	2.24 (1.83, 2.77)	2.02 (1.42, 2.53)	0.013	0.026	0.723	0.058
HDL (mmol/l)	0.99 (0.80, 1.26)	1.03 (0.82, 1.34)	0.93 (0.77, 1.19)	0.221	0.546	1.000	0.119
Ca^2+^ (mmol/l)	2.3 (2.14, 2.43)	2.29 (2.14, 2.42)	2.22 (2.13, 2.35)	0.078	1.000	0.304	0.312
K^+^ (mmol/l)	4.64 (4.33, 5.15)	5.03 (4.57, 5.60)	4.98 (4.43, 5.36)	0.005	0.009	0.369	0.466
Phosphorus (mmol/l)	1.47 (1.26, 1.85)	1.63 (1.42, 1.98)	1.77 (1.50, 2.10)	0.000	0.012	0.005	0.015
iPTH (pmol/l)	29.62 (15.43, 121.20)	32.06 (19.59, 60.10)	37.59 (23.48, 90.62)	0.236	1.000	1.000	1.000
CRP (mg/l)	0.22 (0.10, 0.42)	0.23 (0.1, 0.44)	0.30 (0.12, 0.74)	0.088	1.000	0.078	0.527
Ferritin (ng/ml)	74.40 (35.88, 156.90)	70.2 (26.2, 144.2)	67.4 (29, 126.8)	0.616	1.000	0.745	1.000

Patients were divided into three groups according to UAR tertiles: the low UAR group (UAR <8.70), the middle UAR group (UAR ≥ 8.70 and <11.34), and the high UAR group (UAR ≥ 11.34). Comparison of baseline characteristics among the groups revealed no statistically significant differences, except for hemoglobin, lymphocyte count, total cholesterol, uric acid, albumin, potassium, and phosphorus levels. Compared with the low UAR group, the high UAR group had higher levels of uric acid, potassium, and phosphorus (*P* < 0.05) and lower levels of albumin (*P* < 0.05).

### 3.2 UAR and all-cause mortality

**Figure 2:**
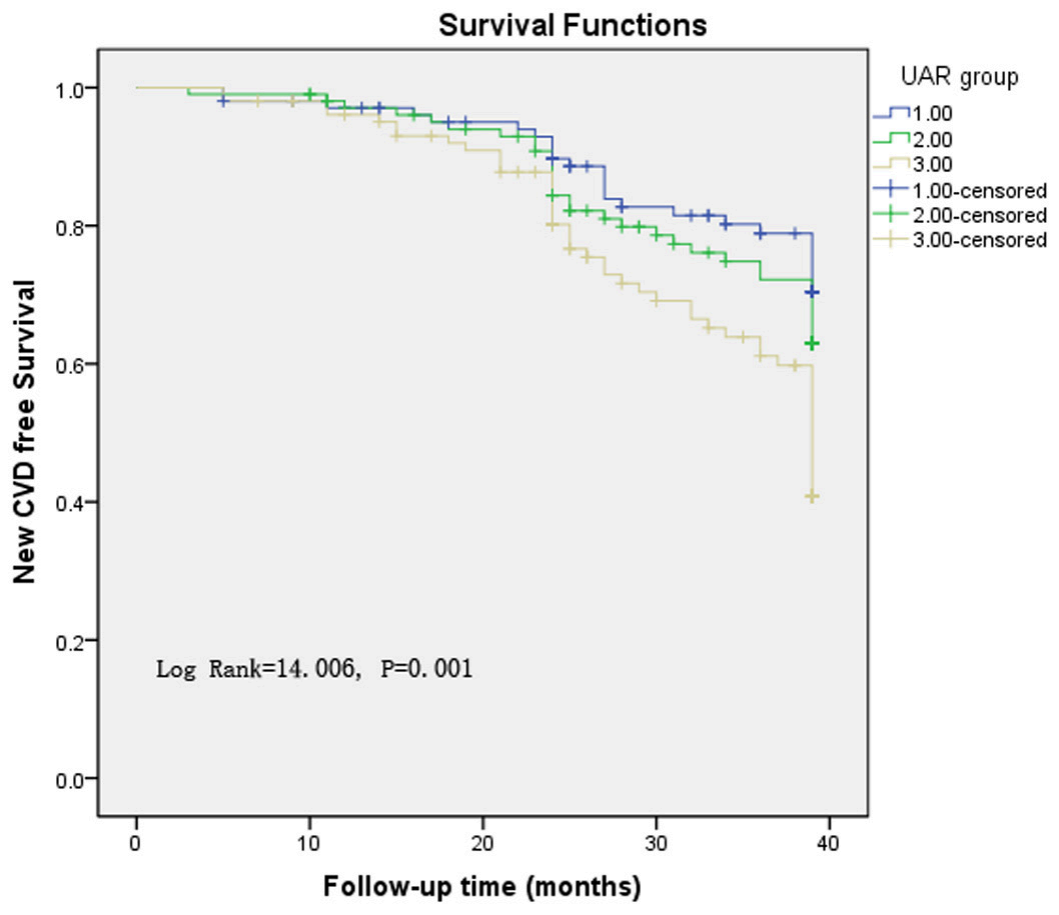
Kaplan–Meier curves for all-cause mortality in patients undergoing hemodialysis according to UAR.

At the end of the follow-up period, 101 patients (32.6%) had died; among them, 35 (34.7%) died from CVD events, 41 (40.6%) from infection, 8 (7.9%) from multi-organ failure, and 17 (16.8%) from unknown causes. Kaplan–Meier survival curves showed significantly higher mortality rates in the middle and high UAR groups than in the low UAR group (log-rank = 10.684, *P* = 0.005) (Figure 2). According to the Cox proportional hazards model, Table 2 showed the associations between baseline characteristics and all-cause mortality. Age (HR = 1.050, 95% CI = 1.032–1.069, *P* = 0.000), diabetes mellitus (HR = 1.875, 95% CI= 1.266–2.776, *P* = 0.002), history of coronary artery disease (HR = 1.562, 95% CI = 1.056–2.309, *P* = 0.025), uric acid (HR = 1.002, 95% CI = 1.000–1.003, *P* = 0.033), UAR (HR = 2.303, 95% CI = 1.369–3.874, *P* = 0.002), iPTH (HR = 0.997, 95% CI= 0.995–1.000, *P* = 0.021), and CRP (HR = 1.523, 95% CI = 1.234–1.880, *P* = 0.000) were associated with an increased risk of mortality. Albumin levels (HR = 0.931, 95% CI = 0.896–0.968, *P* = 0.000) were negatively associated with all-cause mortality. UAR was associated with an increased risk of all-cause mortality in hemodialysis patients (HR: 2.303, 95%; CI: 1.369–3.874, *p* = 0.002), and this association remained statistically significant after adjustment for confounders including age, diabetes, coronary artery disease, iPTH, and CRP (HR: 2.431, 95%; CI: 1.407–4.200, *p* = 0.001) (Table 3).

**Table 2:** Univariate Cox analysis of clinical outcomes in patients undergoing hemodialysis.

Variables	All-cause mortality		New-onset CVD events	
	Univariate analysis		Univariate analysis	
	HR (95% CI)	*P-*value	HR (95% CI)	*P-*value
Gender (male)	0.755 (0.501, 1.138)	0.179	0.975 (0.675, 1.466)	0.980
Age	1.050 (1.032, 1.069)	0.000	1.047 (1.029, 1.065)	0.000
BMI (kg/m^2^)	0.971 (0.917, 1.029)	0.320	0.978 (0.926, 1.032)	0.411
Diabetes	1.875 (1.266, 2.776)	0.002	1.998 (1.359, 2.936)	0.000
Hypertension	0.531 (0.168, 1.675)	0.331	0.756 (0.186, 3.066)	0.695
Coronary artery disease (*n*, %)	1.562 (1.056, 2.309)	0.025	2.702 (1.805, 4.403)	0.000
White blood cell count (× 109/L)	1.014 (0.924, 1.112)	0.768	1.024 (0.936, 1.120)	0.605
Hemoglobin (g/L)	0.997 (0.987, 1.007)	0.605	0.997 (0.987, 1.007)	0.525
Platelet count (× 109/L)	0.999 (0.996, 1.002)	0.581	1.001 (0.998, 1.003)	0.665
Glucose (mmol/l)	1.04 (0.995, 1.087)	0.08	1.066 (1.024, 1.109)	0.002
Albumin (g/L)	0.931 (0.896, 0.968)	0.000	0.920 (0.885, 0.956)	0.000
Uric acid (umol/l)	1.002 (1.000–1.003)	0.033	1.001 (0.999, 1.003)	0.228
UAR (>11.34)	2.303 (1.369,3.874)	0.002	2.278 (1.404, 3.696)	0.001
Total cholesterol	0.944 (0.778, 1.446)	0.559	0.885 (0.732, 1.070)	0.208
Triglyceride	0.935 (0.777, 1.125)	0.476	0.999 (0.859, 1.161)	0.985
HDL (mmol/l)	0.948 (0.737, 1.220)	0.679	0.768 (0.447, 1.319)	0.339
LDL (mmol/l)	1.005 (0.574, 1.760)	0.985	0.870 (0.682, 1.110)	0.263
Phosphorus (mmol/l)	0.757 (0.524, 1.091)	0.757	0.751 (0.527, 1.069)	0.111
iPTH (pmol/l)	0.997 (0.995, 1.000)	0.021	1.000 (0.998, 1.001)	0.804
CRP (mg/l)	1.523 (1.234, 1.880)	0.000	1.503 (1.175, 1.922)	0.001
Ferritin (ng/ml)	0.999 (0.997, 1.001)	0.302	0.999 (0.997, 1.001)	0.400

**Table 3:** Multivariate Cox analysis of all-cause mortality in patients undergoing hemodialysis.

Variables	Multivariate analysis		VIF
	HR (95% CI)	*P-*value	
Age	1.040 (1.020, 1.060)	0.000	1.632
Diabetes	1.753 (1.163, 2.642)	0.007	2.514
Coronary artery disease (*n*, %)	1.211 (0.799, 1.835)	0.367	1.276
UAR (>11.34)	2.431 (1.407, 4.200)	0.001	1.65**0**
iPTH (pmol/l)	0.997 (0.994, 1.000)	0.045	1.531
CRP (mg/l)	1.405 (1.126, 1.754)	0.003	1.365

### 3.3 UAR and new-onset CVD events

**Figure 3:**
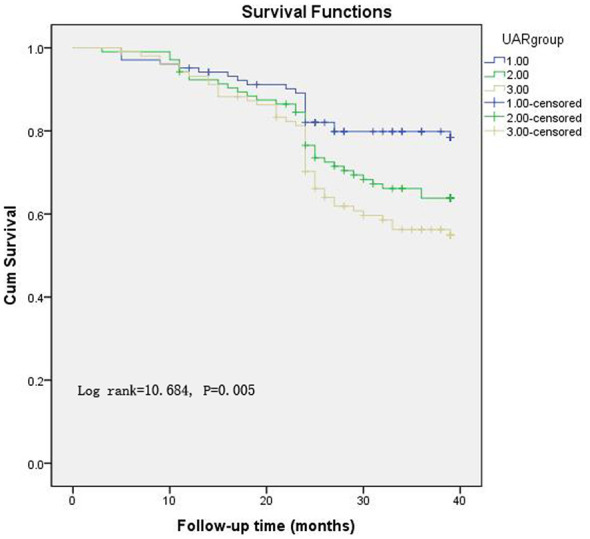
Kaplan–Meier curves for new-onset CVD events in patients undergoing hemodialysis according to URA.

During the 40-month follow-up period, 105 patients (33.9%) occurred new-onset CVD events. The Kaplan–Meier curve comparing CVD events was shown in Figure 3. The incidence of CVD events was significantly higher in the high UAR group (Log rank= 14.006, *P* = 0.001). Results of the univariate Cox regression models used to study the risk factors for CVD events were shown in Table 2. Age (HR = 1.047, 95% CI = 1.029–1.065, *P* = 0.000), diabetes mellitus (HR = 1.998, 95% CI = 1.359–2.936, *P* = 0.000), history of coronary artery disease (HR = 2.702, 95% CI= 1.805–4.403, *P* = 0.000), UAR (HR = 2.278, 95% CI = 1.404–3.696, *P* = 0.001), and CRP (HR = 1.503, 95% CI = 1.175–1.922, *P* = 0.001) were associated with an increased incidence of CVD events. Albumin (HR = 0.920, 95% CI = 0.885–0.956, *P* = 0.000) was negatively associated with CVD events. UAR was associated with an increased risk of CVD events in patients undergoing hemodialysis (HR: 2.303, 95%; CI: 1.369–3.874, *p* = 0.002). High UAR (HR = 2.278, 95% CI= 1.404–3.696, *P* = 0.001) remained an independent risk factor for new-onset CVD events after adjustment for cofounders, including age, diabetes, coronary artery disease, and CRP (HR: 2.163, 95%; CI: 1.304–3.588, *P* = 0.003) (Table 4).

**Table 4:** Multivariate Cox analysis of new-onset CVD events in patients undergoing hemodialysis.

Variables	Univariate analysis		VIF
	HR (95% CI)	*P-*value	
Age	1.043 (1.023, 1.063)	0.000	1.334
Diabetes	1.800 (1.146, 2.826)	0.011	1.850
Coronary artery disease (*n*, %)	2.124 (1.374, 3.281)	0.001	1.725
Glucose (mmol/l)	1.017 (0.964, 1.071)	0.541	1.227
UAR (>11.34)	2.163 (1.304, 3.588)	0.003	1.702
CRP (mg/l)	1.422 (1.092, 1.852)	0.009	1.439

## 4 Discussion

To our knowledge, this is the first study to demonstrate that a high UAR is an independent risk factor for all-cause mortality and CVD events, even after adjustment for traditional risk factors such as age, diabetes mellitus, coronary artery disease, uric acid, albumin, and CRP. In this retrospective cohort study, our findings indicate that UAR, as a composite biomarker integrating inflammatory, metabolic, and nutritional pathways, may serve as a reliable predictor of all-cause mortality and CVD events in patients undergoing hemodialysis. These results also highlight the potential benefits of managing nutritional status and hyperuricemia.

CVD is the primary cause of death in patients receiving maintenance dialysis, and the mortality rate due to CVD in patients undergoing hemodialysis is approximately ten times higher than that in the general population (15, 16). In our cohort, CVD events (34.7%) and infection (40.6%) were the main causes of death in those undergoing maintenance dialysis. Hyperuricemia and serum albumin levels are two important risk factors for CVD in patients with hemodialysis (17, 18). Although contributes 30%−50% of the body's antioxidant capacity and serves as a nutritional marker (19, 20), some studies have found that both low and high uric acid levels are associated with increased mortality in patients undergoing hemodialysis. Nevertheless, improved nutritional status appears to have a greater impact on long-term survival than elevated uric acid levels alone (21–23). Thus, the mortality and cardiovascular risk in dialysis cannot be accurately predicted using uric acid alone. Albumin, a negative acute phase reactant, has antioxidant and anti-inflammatory properties and participates in multiple biological processes (24). The reason for the inconsistent association between UAR and all-cause mortality in HD patients remains unclear, potentially because UAR may reflect a balance between oxidative stress and nutritional status. Both nutritional status and oxidative stress are key regulators of inflammasome activation, and inflammation is known to play a central role in the pathophysiology of atherosclerosis, which represents the most prevalent cause of poor survival in HD patients (25), then UAR may reflect the systemic inflammation state of the body. Li et al. (26) showed that UAR was independently associated with long-term cardiac mortality in patients with unstable angina pectoris receiving percutaneous coronary intervention. Duman et al. (14) found that UAR, a marker of inflammation, was an independent predictor of preprocedural intracoronary artery thrombus in patients with STEMI. Another study reported that in patients with diabetes, UAR was associated with all-cause and cardiovascular death and had a higher predictive value than uric acid alone (27). The UAR had also been investigated in kidney diseases. Ertan et al. (28) found that UAR was linked to both AKI and death in patients in intensive care units. Özgür et al. (29) reported that in patients with AKI, UAR was related to short-term mortality. Moreover, Saylik et al. (30) revealed that in predicting the development of contrast-induced nephropathy (CIN), UAR was more predictive than serum uric acid and albumin. To the best of our knowledge, this is the first study to investigate the relationship between UAR, all-cause mortality, and CVD events in patients undergoing maintenance hemodialysis. In our study, the results showed that the mortality rates and the incidence of CVD events in the middle and high UAR groups were significantly higher than those in the low UAR group (Log rank = 10.684, *P* = 0.005, Log rank = 14.006, *P* = 0.001), demonstrating that high UAR level was an independent risk factor for all-cause mortality and CVD events, even after adjustment for traditional risk factors. The mechanisms underlying this association remain unclear; however, one possible explanation is that UAR reflects the combined effects of oxidative stress and nutritional status, which are important modulators of inflammation in this population (7, 17, 31–33).

However, our study had some limitations. First, its retrospective, single-center design and limited sample size may introduce selection bias. Second, the relatively short follow-up period prevented assessment of the long-term predictive value of UAR. Third, potential unmeasured confounders may have influenced the observed associations, such as serum creatinine, urea, the use of urate-lowering medication, dialysis vintage, dialysis adequacy, and vascular access type. Competing risks were not considered in the Cox regression analysis due to the limited number of competing events, which precluded a stable competing-risk model. Finally, UAR was assessed only at baseline, and longitudinal changes were not evaluated. Therefore, large-scale, multicenter prospective studies with longer follow-up durations are warranted.

## 5 Conclusions

In this study, we demonstrated for the first time that UAR was independently and significantly associated with all-cause mortality and CVD events in patients undergoing hemodialysis. These findings suggest that UAR may serve as a valuable composite biomarker integrating inflammatory, metabolic, and nutritional pathways, and may provide new insights into improving hemodialysis outcomes. Further research is needed to clarify the clinical utility of UAR and to guide patient management strategies.

## Data Availability

The raw data supporting the conclusions of this article will be made available by the authors, without undue reservation.

## References

[1] Jassal SV Karaboyas A Comment LA Bieber BA Morgenstern H Sen A . Functional dependence and mortality in the international dialysis outcomes and practice patterns study (DOPPS). Am J Kidney Dis. (2016) 67:283–92. doi: 10.1053/j.ajkd.2015.09.024 26612280 PMC5530761

[2] Cozzolino M Mangano M Stucchi A Ciceri P Conte F Galassi A. Cardiovascular disease in dialysis patients. Nephrol Dial Transplant. (2018) 33:iii28–34. doi: 10.1093/ndt/gfy174 30281132 PMC6168816

[3] Li ZL He CS Chen YH Liang XL Dong W Li RZ . Association of heart valve calcification with cardiovascular outcomes in patients on maintenance hemodialysis. Nan Fang Yi Ke Da Xue Xue Bao. (2016) 36:941–6. 27435773

[4] Yang Y Qin X Li Y Yang S Chen J He Y . Relationship between serum uric acid and mortality risk in hemodialysis patients: a multicenter prospective cohort study. Am J Nephrol. (2020) 51:823–32. doi: 10.1159/000509258 33070128

[5] Hsu SP Pai MF Peng YS Chiang CK Ho TL Hung KY. Serum uric acid levels show a ‘J-shaped' association with all-cause mortality in haemodialysis patients. Nephrol Dial Transplant. (2004) 19:457–62. doi: 10.1093/ndt/gfg563 14736974

[6] Lee SM Lee AL Winters TJ Tam E Jaleel M Stenvinkel P . Low serum uric acid level is a risk factor for death in incident hemodialysis patients. Am J Nephrol. (2009) 29:79–85. doi: 10.1159/000151292 18689987 PMC2786018

[7] Beberashvili I Erlich A Azar A Sinuani I Feldman L Gorelik O . Longitudinal study of serum uric acid, nutritional status, and mortality in maintenance hemodialysis patients. Clin J Am Soc Nephrol. (2016) 11:1015–23. doi: 10.2215/CJN.10400915 27026520 PMC4891753

[8] Beberashvili I Sinuani I Azar A Shapiro G Feldman L Stav K . Serum uric acid as a clinically useful nutritional marker and predictor of outcome in maintenance hemodialysis patients. Nutrition. (2015) 31:138–47. doi: 10.1016/j.nut.2014.06.012 25466658

[9] Park C Obi Y Streja E Rhee CM Catabay CJ Vaziri ND . Serum uric acid, protein intake and mortality in hemodialysis patients. Nephrol Dial Transplant. (2017) 32:1750–7. doi: 10.1093/ndt/gfw419 28064158 PMC5837687

[10] Altomare AA Brioschi M Eligini S Bonomi A Zoanni B Iezzi A . N-acetylcysteine regenerates *in vivo* mercaptoalbumin. Antioxidants. (2022) 11:1758. doi: 10.3390/antiox11091758 36139832 PMC9495570

[11] Chien SC Chandramouli C Lo CI Lin CF Sung KT Huang WH . Associations of obesity and malnutrition with cardiac remodeling and cardiovascular outcomes in Asian adults: a cohort study. PLoS Med. (2021) 18:e1003661. doi: 10.1371/journal.pmed.1003661 34061848 PMC8205172

[12] Chen JB Cheng BC Yang CH Hua MS. An association between time-varying serum albumin level and the mortality rate in maintenance haemodialysis patients: a five-year clinical cohort study. BMC Nephrol. (2016) 17:117. doi: 10.1186/s12882-016-0332-5 27542730 PMC4992318

[13] Uludag K Boz G Gunal AI. Lower serum albumin level is associated with increased risk of hospital admission and length of stay in hospital among incident hemodialysis patients by using overdispersed model. Ther Apher Dial. (2021) 25:179–87. doi: 10.1111/1744-9987.13552 32584500

[14] Duman H Ipek E Durak H Sahin MA Ergül E Yilmaz AS . Uric acid to albumin ratio as a predictive marker for intracoronary thrombus severity in ST-segment elevation myocardial infarction (STEMI) patients undergoing primary percutaneous coronary intervention (PCI). Med Sci Monit. (2024) 30:e945832. doi: 10.12659/MSM.945832 39425464 PMC11497856

[15] National Kidney Foundation. K/DOQI clinical practice guidelines for chronic kidney disease: evaluation, classification, and stratification. Am J Kidney Dis. (2002) 39:S1–266. 11904577

[16] de Jager DJ Grootendorst DC Jager KJ van Dijk PC Tomas LM Ansell D . Cardiovascular and noncardiovascular mortality among patients starting dialysis. JAMA. (2009) 302:1782–9. doi: 10.1001/jama.2009.1488 19861670

[17] Mehrotra R Duong U Jiwakanon S Kovesdy CP Moran J Kopple JD . Serum albumin as a predictor of mortality in peritoneal dialysis: comparisons with hemodialysis. Am J Kidney Dis. (2011) 58:418–28. doi: 10.1053/j.ajkd.2011.03.018 21601335 PMC3159826

[18] Zawada AM Carrero JJ Wolf M Feuersenger A Stuard S Gauly A . Serum uric acid and mortality risk among hemodialysis patients. Kidney Int Rep. (2020) 5:1196–206. doi: 10.1016/j.ekir.2020.05.021 32775819 PMC7403560

[19] Feig DI Kang D Johnson RJ. Uric acid and cardiovascular risk. N Engl J Med. (2008) 359:1811–21. doi: 10.1056/NEJMra0800885 18946066 PMC2684330

[20] Crawley WT Jungels CG Stenmark KR Fini MA. U-shaped association of uric acid to overall-cause mortality and its impact on clinical management of hyperuricemia. Redox Biol. (2022) 51:102271. doi: 10.1016/j.redox.2022.102271 35228125 PMC8889273

[21] Gan W Zhu F Fang X Wang W Shao D Mao H . Association between serum uric acid and all-cause and cardiovascular-related mortality in hemodialysis patients. Front Nutr. (2024) 11:1499438. doi: 10.3389/fnut.2024.1499438 39686955 PMC11646772

[22] Xia X He F Wu X Peng F Huang F Yu X. Relationship between serum uric acid and all-cause and cardiovascular mortality in patients treated with peritoneal dialysis. Am J Kidney Dis. (2014) 64:257–64. doi: 10.1053/j.ajkd.2013.08.027 24176223

[23] Jeon JS Chung SH Han DC Noh H Kwon SH Lindholm B . Mortality predictive role of serum uric acid in diabetic hemodialysis patients. J Ren Nutr. (2014) 24:336–42. doi: 10.1053/j.jrn.2014.05.005 25167998

[24] Roche M Rondeau P Singh NR Tarnus E Bourdon E. The antioxidant properties of serum albumin. FEBS Lett. (2008) 582:1783–7. doi: 10.1016/j.febslet.2008.04.057 18474236

[25] Song S Cai X Hu J Zhu Q Shen D Heizhati M . Correlation between plasma aldosterone concentration and bone mineral density in middle-aged and elderly hypertensive patients: potential impact on osteoporosis and future fracture risk. Front Endocrinol. (2024) 15:1373862. doi: 10.3389/fendo.2024.1373862 38808106 PMC11130431

[26] Li S Chen H Zhou L Cui H Liang S Li H. The uric acid to albumin ratio: a novel predictor of long-term cardiac mortality in patients with unstable angina pectoris after percutaneous coronary intervention. Scand J Clin Lab Invest. (2022) 82:304–10. doi: 10.1080/00365513.2022.2084698 35675042

[27] Chen S Zhang M Hu S Shao X Liu L Yang Z . Uric acid to albumin ratio is a novel predictive marker for all-cause and cardiovascular death in diabetic patients: a prospective cohort study. Front Endocrinol. (2024) 15:1388731. doi: 10.3389/fendo.2024.1388731 PMC11794066 39911231

[28] Ertan OES Gökçe O Bal C Kocaturk E Ertan O Mutluay R. Investigation of the relationship between serum uric acid-to-albumin ratio and 28-day mortality in patients with and without acute kidney injury. J Acute Med. (2024) 14:152–9. doi: 10.1016/j.jcrc.2024.154607 39624148 PMC11608859

[29] Özgür Y Akin S Yilmaz NG Gücün M Keskin Ö. Uric acid albumin ratio as a predictive marker of short-term mortality in patients with acute kidney injury. Clin Exp Emerg Med. (2021) 8:82–8. doi: 10.15441/ceem.20.024 34237812 PMC8273677

[30] Saylik F Çinar T Akbulut T Selçuk M. Serum uric acid to albumin ratio can predict contrast-induced nephropathy in ST-elevation myocardial infarction patients undergoing primary percutaneous coronary intervention. Angiology. (2023) 74:70–8. doi: 10.1177/00033197221091605 35451314

[31] Stepanova N Korol L Ostapenko T Marchenko V Belousova O Snisar L . Pre-infection nutritional status, oxidative stress, and one-year-long COVID persistence in patients undergoing hemodialysis: a prospective cohort study. Clin Pract. (2024) 14:892–905. doi: 10.3390/clinpract14030070 38804402 PMC11130966

[32] Ilic Begovic T Radic J Radic M Modun D Seselja-Perisin A Tandara L. Seasonal variations in nutritional status and oxidative stress in patients on hemodialysis: are they related? Nutrition. (2021) 89:111205. doi: 10.1016/j.nut.2021.111205 33836426

[33] Shen T Jiang L Zhang Q Xv M Wu S. Effect of oral nutritional supplements on inflammation and oxidative stress in hemodialysis patients: a meta-analysis. Int Urol Nephrol. (2025) 57:2547–56. doi: 10.1007/s11255-025-04427-z 39992551

