# Supplementary material for: Serum uric acid-to-albumin ratio as a novel predictor of all-cause mortality and cardiovascular events in hemodialysis patients: a retrospective cohort study
Source: Front Nutr. 2026 Jul 14;13:1818356. doi: 10.3389/fnut.2026.1818356 (PMC13408410; doi:10.3389/fnut.2026.1818356)
Supplement: Supplementary file 1 [file Presentation_1.pptx]

## Slide 1
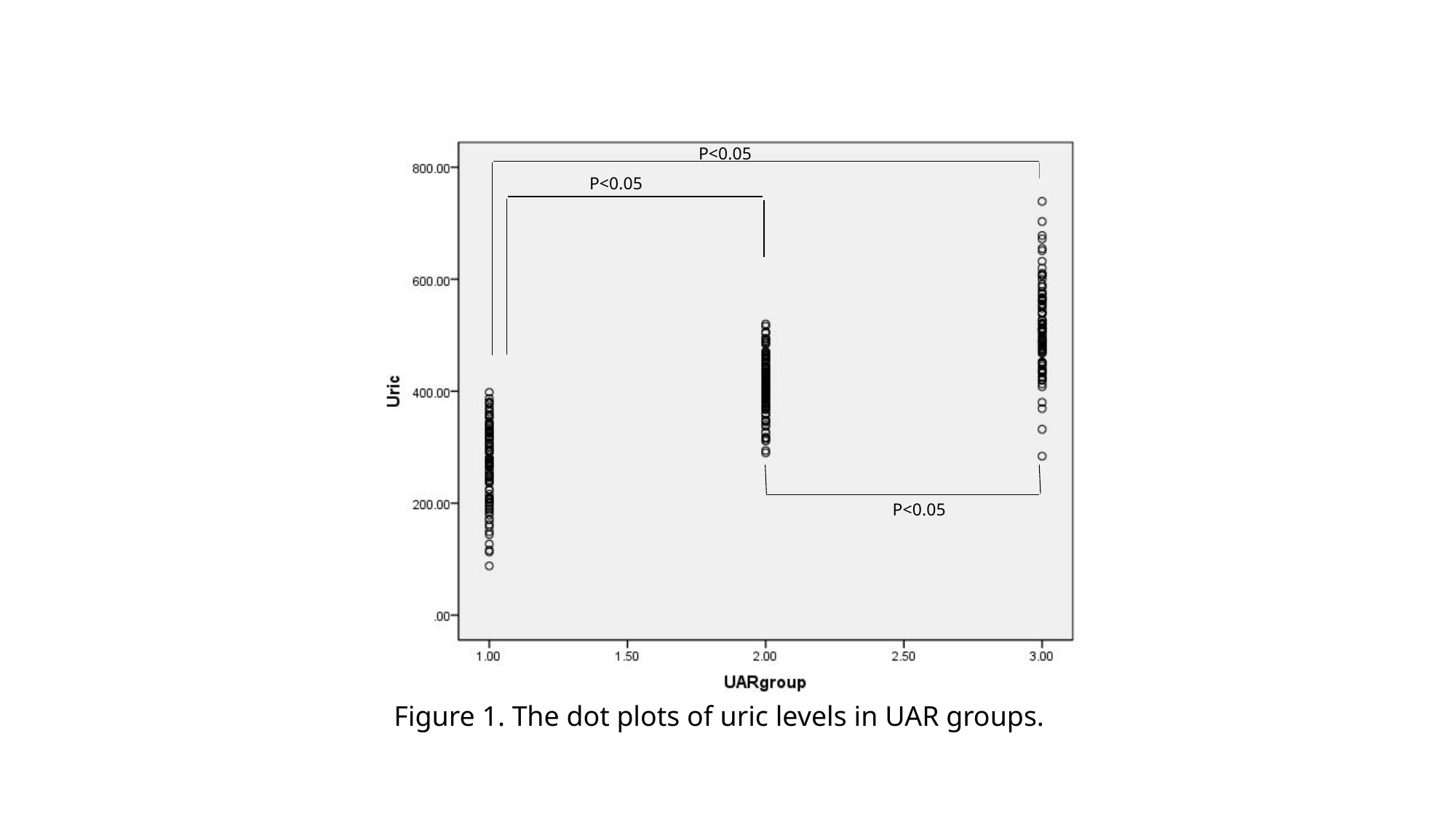

P<0.05
P<0.05
P<0.05
Figure 1. The dot plots of uric levels in UAR groups.

## Slide 2
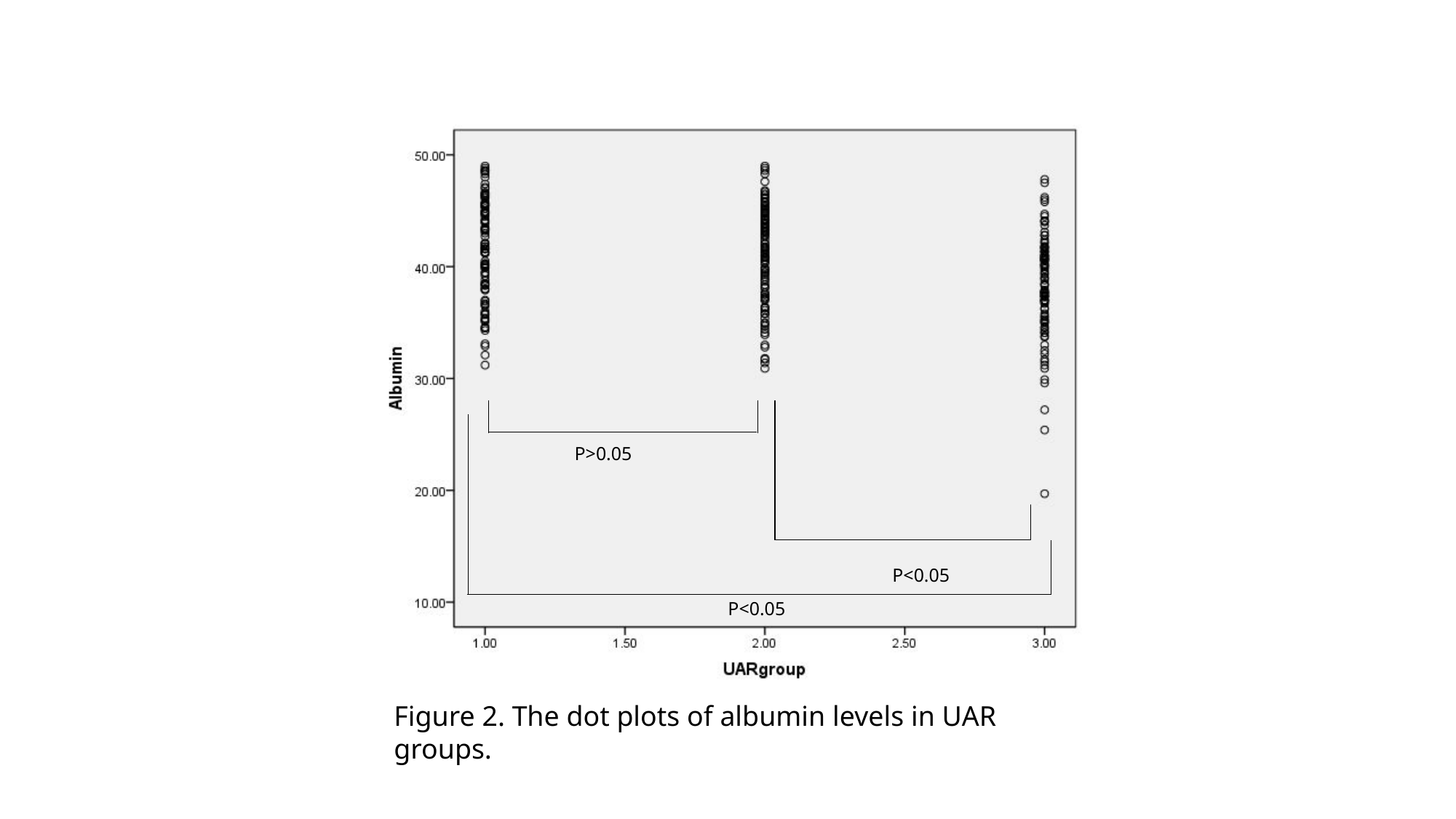

P>0.05
P<0.05
P<0.05
Figure 2. The dot plots of albumin levels in UAR groups.
